# Methods to improve the quality of smoking records in a primary care EMR database: exploring multiple imputation and pattern-matching algorithms

**DOI:** 10.1186/s12911-020-1068-5

**Published:** 2020-03-14

**Authors:** Stephanie Garies, Michael Cummings, Hude Quan, Kerry McBrien, Neil Drummond, Donna Manca, Tyler Williamson

**Affiliations:** 1 Department of Family Medicine, University of Calgary, G012 Health Sciences Centre, 3330 Hospital Drive NW, Calgary, Alberta T2N 4N1 Canada; 2 Department of Community Health Sciences, University of Calgary, 3280 Hospital Drive NW, Calgary, Alberta T2N 4Z6 Canada; 3 Department of Family Medicine, University of Alberta, 6-10 University Terrace, Edmonton, Alberta T6G 2T4 Canada; 4 School of Public Health, University of Alberta, 3-300 Edmonton Clinic Health Academy, 11405-87 Ave, Edmonton, Alberta T6G 1C9 Canada

**Keywords:** Electronic medical records, Primary health care, Public health informatics, Smoking

## Abstract

**Background:**

Primary care electronic medical record (EMR) data are emerging as a useful source for secondary uses, such as disease surveillance, health outcomes research, and practice improvement. These data capture clinical details about patients’ health status, as well as behavioural risk factors, such as smoking. While the importance of documenting smoking status in a healthcare setting is recognized, the quality of smoking data captured in EMRs is variable. This study was designed to test methods aimed at improving the quality of patient smoking information in a primary care EMR database.

**Methods:**

EMR data from community primary care settings extracted by two regional practice-based research networks in Alberta, Canada were used. Patients with at least one encounter in the previous 2 years (2016–2018) and having hypertension according to a validated definition were included (*n* = 48,377). Multiple imputation was tested under two different assumptions for missing data (smoking status is missing at random and missing not-at-random). A third method tested a novel pattern matching algorithm developed to augment smoking information in the primary care EMR database. External validity was examined by comparing the proportions of smoking categories generated in each method with a general population survey.

**Results:**

Among those with hypertension, 40.8% (*n* = 19,743) had either no smoking information recorded or it was not interpretable and considered missing. Those with missing smoking data differed statistically by demographics, clinical features, and type of EMR system used in the clinic. Both multiple imputation methods produced fully complete smoking status information, with the proportion of current smokers estimated at 25.3% (data missing at random) and 12.5% (data missing not-at-random). The pattern-matching algorithm classified 18.2% of patients as current smokers, similar to the population-based survey (18.9%), but still resulted in missing smoking information for 23.6% of patients. The algorithm was estimated to be 93.8% accurate overall, but varied by smoking status category.

**Conclusion:**

Multiple imputation and algorithmic pattern-matching can be used to improve EMR data post-extraction but the recommended method depends on the purpose of secondary use (e.g. practice improvement or epidemiological analyses).

## Background

There has been a transformative shift towards the uptake of electronic medical record (EMR) systems in primary care settings and the subsequent re-use of these data for a variety of secondary purposes. In Canada, the Canadian Primary Care Sentinel Surveillance Network (CPCSSN) has established a source of de-identified, patient-level primary care EMR data that has been used for health research, disease surveillance, and quality improvement [1]. This has also included the development of extensive processes and algorithms to extract, transform, and standardize the raw EMR data generated from nearly 2 million patients in 7 Canadian provinces or territories [1, 2].

One distinct advantage of the CPCSSN data is the availability of behavioural risk factor information (such as smoking, alcohol use, diet and exercise) coupled with clinically-verified health measures and outcomes. However, risk factor data are often entered in unstructured fields or as free text in different areas of the EMR, which also varies by the type of EMR system being used. This creates difficulties during the data extraction process, as well as analytic challenges for data users, and also impacts the quality of these data in terms of completeness and accuracy. For instance, more than one-third of all patients over 16 years of age in the national CPCSSN database were missing smoking information, with biases likely introduced due to how smoking status was captured and for whom [3].

When conducting observational studies using EMR data, it is not uncommon to encounter missing data. Part of the challenge is determining whether the data are missing at random (where the probability of missingness depends on other observed information or data) or missing not-at-random (where the probability of missingness depends on unobserved or unknown information) [4], in addition to deciding on the most appropriate technique to use for analysis when data are missing. For some situations, it may not be recommended to use a complete case analysis (i.e. including only those who do not have missing data), as this method can reduce sample size and statistical power, lower precision of results, and produce biased findings [4]. Multiple imputation (MI) has been touted as a more accurate and less biased method, as missing data are imputed based on the distributions and variability of other data elements in the sample [4]. The use of MI to improve the recording of smoking data has been previously demonstrated in a large U.K. general practitioner database, where the authors observed that smoking data were likely missing non-randomly and had identified potential misclassification of ex-smokers who were recorded as non-smokers, particularly if a substantial period of time had passed since quitting or if quitting occurred at a younger age [5, 6].

The objective of this study is to explore methods aimed at improving the completeness of patient smoking status in the CPCSSN database, specifically focused on patients with hypertension. Two methods will test MI using different scenarios for data missing at random (MAR) or missing not at random (MNAR). The third method will test a newly-developed CPCSSN-based classifier for assigning individual smoking status based on additional free text in the smoking record.

## Methods

### Data source

This study used CPCSSN data collected by two practice-based research networks in Alberta, Canada (Northern Alberta Primary Care Research Network [NAPCReN], Southern Alberta Primary Care Research Network [SAPCReN]). Over 320 community-based providers (mostly family physicians with a small proportion of nurse practitioners and pediatricians) contribute de-identified EMR data from close to 400,000 patients across Alberta. Healthcare is publicly funded in Canada and typically administered by each province or territory. There is no mandatory patient registration in primary care, although patient rostering is increasingly undertaken in Alberta.

The CPCSSN data contain nearly all information entered into the patient EMR, including demographics, patient profile (e.g., medical diagnoses, history), physical measurements (e.g., blood pressure, height, weight), risk factor information, laboratory results, prescribed medications, medical procedures, referrals, and physician billing claims [1]. Additional details about the regional extraction, processing and transformation for NAPCReN and SAPCReN have been described elsewhere [7].

### Sample

This analysis focused on a cohort of patients with hypertension, as guidelines recommend that tobacco use status should be updated on a regular basis for these patients [8]. Patients were identified as hypertensive if they met the validated definition developed by CPCSSN, which includes a combination of diagnostic codes related to hypertension and antihypertensive medications [9]. This definition demonstrated good validity as compared to the reference standard chart review (sensitivity = 84.9%; specificity = 93.5%) [9]. Patients with at least one encounter with their primary care provider within the previous 2 years (July 1, 2016 to June 30, 2018) were included; this is considered an acceptable indicator of ‘active’ patients in a practice, as the large majority of individuals visit a family physician at least once in a two-year period [10]. Those who were explicitly recorded as deceased or inactive according to their EMR status were excluded. Data from the entire history of the patient EMR were used for the analysis; the earliest timepoint varies by patient and depends on when a clinic integrated their EMR system into practice and when the patient first attended the clinic.

### Defining smoking status

The smoking record for each patient found in the Risk Factor table of the CPCSSN database was used. Patient smoking status can be first documented at any time and updated as often or as infrequently as chosen by the patient and provider. For those patients who had more than one smoking status documented at any time throughout the history of their EMR, the most recent status was used according to the date the record was created in the clinic EMR system. Each risk factor record is comprised of several fields including: a unique CPCSSN identifier for each patient, date on which the record was entered into the EMR, risk factor name, risk factor status, and risk factor values (i.e. any quantitative or extraneous information). Records with the risk factor name coded as ‘Smoking’ or ‘Unknown’ were selected for this analysis. The ‘Status’ of these risk factor records contained the original EMR data and consisted of four categories: current, past, never, and unknown. For the imputation, all unknown statuses were recoded as missing data.

### Defining demographic and clinical variables

The relevant patient characteristics and clinical values included in the multiple imputation were sex, age, rural or urban residence, body mass index (BMI), systolic blood pressure, diabetes, number of clinical encounters, and type of EMR system used in the practice. Patient residence was considered rural if the second character of the postal code was 0 and urban if the second character was any digit except 0. Most recently recorded blood pressure and body mass index (BMI) were used. BMI was included if it was deemed to be within a plausible range (between 12 and 70 kg/m^2^) and if the difference between subsequent per-patient measurements was within 10 kg/m^2^. Patients were indexed with diabetes and/or chronic obstructive pulmonary disease (COPD) according to validated CPCSSN definitions. The diabetes definition had a sensitivity of 95.6% and specificity of 97.1%; COPD demonstrated a sensitivity of 82.1% and specificity of 97.3% [9]. The number of clinical encounters was a sum of visit dates documented in the EMR for each patient. The earliest patient encounter date in the CPCSSN database varies, depending on when a clinic first implemented an EMR system and when the patient first attended.

### Methods for improving completeness

Three methods for improving the completeness of smoking data were tested. The first two utilized multiple imputation (MI) – this is an analytical technique for improving missing data and can be readily implemented using currently available statistical software (e.g. R). MI first generates many different datasets with the missing values imputed based on the distribution of missing data in the original dataset; then, analyses are conducted on each dataset separately and lastly, the results are pooled to generate a multiple imputed estimate [4]. The advantage of MI is that it allows uncertainty in the missing data to be accounted for and has been shown to result in more accurate standard errors and less biased outcomes compared to a complete case analysis [4].

MI was applied under two different missing data assumptions, similar to Marston et al.’s previous work using a UK general practice EMR database [6]. The first MI method considered smoking status to be ‘missing at random’ (MAR) and missing data for three smoking categories, current, past, and never, were imputed. The second MI method assumes that all current smokers have been recorded (given the importance of smoking as a significant risk factor for hypertensive patients) and thus, smoking status is ‘missing-not-at-random’ (MNAR). Here, only the categories of past or never smokers will be imputed for those missing smoking status data.

The third method used a classifier algorithm recently developed by a CPCSSN data manger (MC). The raw EMR data that are used to create the CPCSSN national risk factor data set, comprising approximately 1 million smoking records in both official languages (English and French), were used to develop text-matching patterns for calculating patient smoking status. These raw data are broken into three text fields: original Name, original Value (e.g., 10 packs/day), and original Status, where ‘original’ refers to the raw data from the EMR. For records that are coded as ‘smoking’, risk factor status is coded to one of the following categories: current, never, past, not current, and unknown. A status of ‘not current’ means it is unclear whether the patient is a past smoker or has never smoked (e.g., ‘non-smoker’). Lastly, records that match either zero or multiple statuses are coded as ‘Unknown’.

**Fig. 1:**
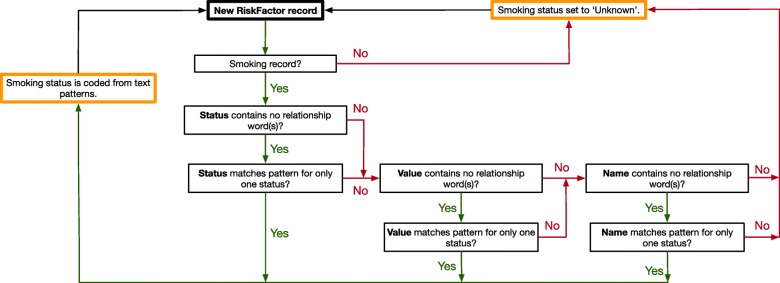
The flow diagram for the pattern-matching smoking status classifier (Method 3)

The smoking status classifier was created following manual review of the top 2000 most frequently occurring smoking records in the national CPCSSN database, comprising approximately 75% of the data. For each record, the true smoking status was determined after review of the text in the all fields related to smoking status in the CPCSSN database (Name, Value, and Status). This text was then used to generate a unique text-pattern that identified the true status. Once all 2000 records were reviewed, a random sample of 1000 records from the remaining 25% of the data set were then analysed to validate and improve pattern-matching accuracy. For each status, a simplified set of all text-patterns, plus additional logic, was then collected to create the smoking status classifier. Figure 1 displays a flow chart of the classifier’s operation. It analyzes each record sequentially by field, with data in the Status field having precedence over Value, and Value over Name, as shown in Fig. 1. Entries that contain relationship words (e.g., aunt, father) are ignored by the classifier, as they are usually family history or other non-risk factor data that typically cause patient smoking status to be misclassified.

### Analysis

Patient demographic and clinical characteristics were reported as proportions for categorical variables, mean with standard deviation (SD) for normally distributed continuous variables, and median with lower and upper quartiles for non-parametric data. Comparisons of characteristics between patients with and without smoking records were conducted using a chi-squared test for categorical variables, one-way test for continuous variables, and Wilcoxon test for non-parametric data.

For the multiple imputation in Method 1 and 2, the MICE package in R was used [11]. This analytic tool uses a fully conditional specification to impute each variable by specifying an imputation model that best suits the variable type. For instance, a Bayesian polytomous regression model was used for the unordered, categorial smoking status data. Forty imputed datasets were generated with 5 iterations taken to impute the missing values.

To obtain error estimates for the MI methods, a simulation was conducted for patients with complete smoking status data. Missing smoking data were randomly introduced for 40.8% of the complete case group (the same proportion of missing smoking status data in the total sample of 48,377).

The classifier algorithm used by Method 3, programmed in Python, was applied to the hypertensive dataset and a ‘calculated’ Status was generated for each smoking record. Method 3 was then validated by manually assigning a smoking status to all unique records, based on the original Status and Value data, while blinded to the result of the algorithmic-calculated Status. Since, for the hypertensive data set, the original Name data only contain one string (‘Smoking’), they were not reviewed during algorithm assessment.

External validity was estimated using Alberta-specific data from the 2017 Canadian Tobacco, Alcohol, and Drugs Survey (CTADS) [12]. CTADS is a biennial cross-sectional telephone survey in Canadians ages 15 years and older conducted by Statistics Canada [12]. Results are available by province and therefore, the smoking status of Alberta respondents was used (*n* = 1444 survey respondents weighted to a population estimate of 3,478,000).

All data analysis was performed in RStudio version 1.1.456.

This study was approved by the Conjoint Health Research Ethics Board at the University of Calgary (REB17–1825) and the Health Research Ethics Board at the University of Alberta (Pro00079372).

## Results

**Fig. 2:**
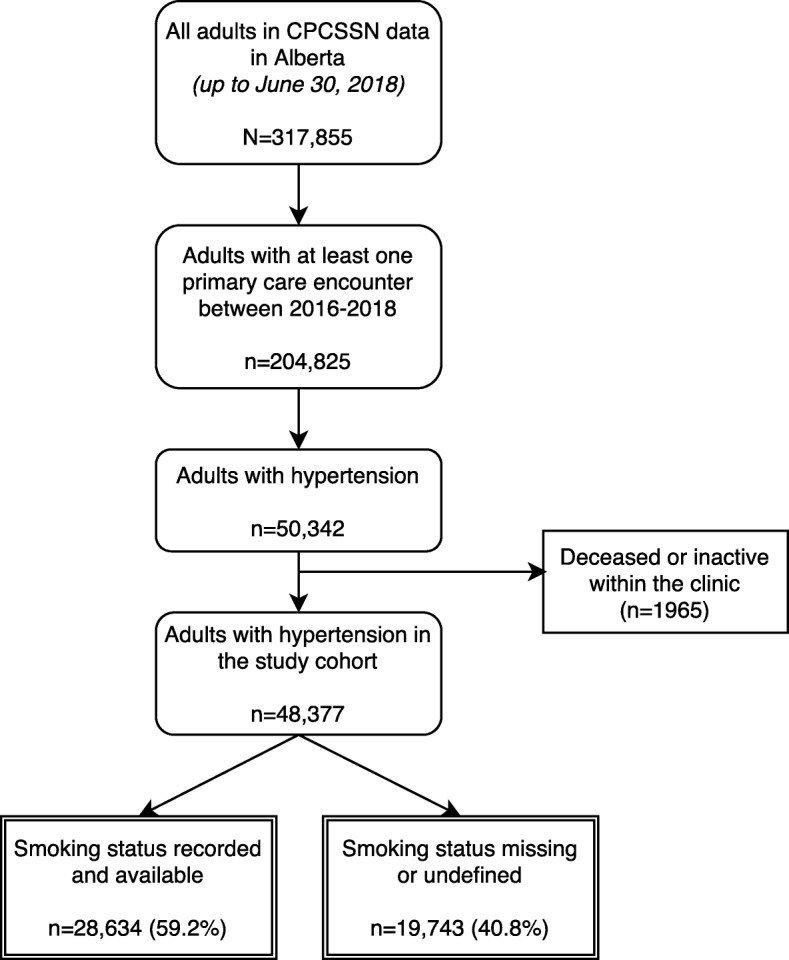
The flow diagram for patients in the study cohort

The flow of patients into the study cohort is summarized in Fig. 2. There were 48,377 adult patients with hypertension identified in the CPCSSN data from Alberta within a two-year contact period who were not deceased or inactive. Of these, 28,634 (59.2%) had a smoking status recorded and available in the database, with approximately 20% having more than one smoking status entry recorded throughout the history of their EMR. For smoking entries with an associated date of entry, nearly 80% were recorded or updated within the previous 2 years (as the ‘most recent’ status was chosen for inclusion). Table 1 summarizes demographic, clinical, and practice characteristics for those with and without smoking information. Those who were missing smoking status information (*n* = 19,743) were more likely to be older (*p* = 0.001), female (*p* = 0.001), have higher systolic (*p* < 0.001) and diastolic (*p* = 0.027) blood pressure, lower BMI (*p* = 0.001), less co-morbid diabetes and COPD (*p* < 0.001), reside in an urban setting (*p* < 0.001), and have fewer total clinical encounters (*p* < 0.001). While the differences in sex, age, BMI, and blood pressure between the two groups were statistically significant, they were small in absolute terms and may not be clinically meaningful.

**Table 1:** Demographic and clinical characteristics of patients with and without smoking status recorded in their EMR

Variable	Smoking status recorded (*N* = 28,634)	Missing smoking status (*N* = 19,743)	***p***-value
Female, n (%)	14,547 (50.8)	10,342 (52.4)	0.001
Missing sex, n (%)	1 (0.0)	1 (0.0)	–
Age in years, mean (SD)	64.9 (13.4)	65.3 (14.7)	0.001
BMI in kg/m^2^, mean (SD)	30.9 (6.8)	30.7 (6.8)	0.001
Missing BMI, n (%)	1660 (5.8)	3334 (16.9)	–
Systolic BP, mean (SD)	132.3 (16.2)	133.4 (16.5)	< 0.001
Diastolic BP, mean (SD)	78.9 (10.8)	79.2 (10.7)	0.027
Missing BP, n (%)	84 (0.3)	406 (2.0)	–
COPD, n (%)	3078 (10.7)	1645 (8.3)	< 0.001
Diabetes, n (%)	8040 (28.1)	4920 (24.9)	< 0.001
Type of EMR system, n (%)			< 0.001
Wolf	20,471 (71.5)	5872 (29.7)	
Med Access	7481 (26.1)	13,110 (66.4)	
Practice Solutions Suite	448 (1.6)	14 (0.1)	
Telin	86 (0.3)	251 (1.3)	
Healthquest	148 (0.5)	496 (2.5)	
Urban residence, n (%)	20,373 (72.6)	15,728 (81.1)	< 0.001
Missing postal code, n (%)	558 (1.9)	344 (1.7)	–
Number of total clinical encounters, median (lower quartile, upper quartile)	37.0 (20, 63)	24.0 (12, 44)	< 0.001

Abbreviations: *BMI* body mass index, *BP* blood pressure, *COPD* chronic obstructive pulmonary disease, *EMR* electronic medical record, *SD* standard deviation

The two groups also differed significantly by the type of EMR system (Wolf, Med Access, Practice Solutions Suite, Healthquest, Telin) used by the primary care practice (*p* < 0.001). Of the five EMR systems included, it was observed that Med Access contributed the largest proportion of patients with missing or undefined smoking data (*n* = 13,110; 66.4%). When examining the missingness of smoking records by each EMR system, Practice Solutions Suite had the most complete (97% of these patients had a smoking record) and Healthquest had the least complete (23% of these patients had a smoking record); however, the total number of patients in these groups were small (462 patients on Practice Solutions EMR; 644 patients on Healthquest EMR).

Lastly, it was also observed that patients who were missing smoking status also had more missing data for other clinical variables (BMI and blood pressure).

### Methods 1 and 2 (multiple imputation)

Table 2 summarizes patient smoking statuses for those without missing data (complete cases), MI for all three statuses (current, past, never; Method 1) and MI for only past or never smokers (Method 2). Both methods resulted in complete data for all patients in the full cohort; however, the proportion of current smokers varied substantially, with 25.3% in Method 1 and 12.5% in Method 2. Compared to the Alberta-specific CTADS survey data [12], Method 1 may be overestimating the proportion of current smokers and Method 2 may be underestimating current smokers. The ‘past’ category for Methods 1 and 2 fall within the survey’s estimated 95% confidence intervals. The relative error for the multiple imputation, which compared the proportions of smoking status in the complete cases to the imputed dataset, was small (current = − 0.05%, past = 0.12%, never = − 0.03%). Any additional free text related to smoking status was deemed to be insufficient for the MI process, as this was rarely available for most patients and was highly unstructured and not easily interpretable (e.g., over 850 unique strings were found with an average of 18 characters within each string; examples of text entries included “smokes socially”, “smoker: quit”, “up to 2ppd”, “yes”, “tobacco use assessment”, etc.).

**Table 2:** Comparison of different improvement methods for smoking categories

Category	2017 CTADS general population survey (Alberta) *N* = 1444	CPCSSN hypertensive patients (Alberta) *N* = 48,377			
	Survey prevalence % (95% CI)	Complete cases n (%)	Method 1: MAR imputation^a^ n (%)	Method 2: MNAR imputation^b^ n (%)	Method 3: CPCSSN pattern matching n (%)
Current Smoker	18.9 (14.8–22.9)	6061 (21.2)	12,255 (25.3)	6061 (12.5)	6729 (18.2)
Past/Former Smoker	21.1 (17.0–25.1)	6494 (22.7)	9628 (19.9)	11,305 (23.4)	8868 (24.0)
Never Smoker	60.1 (55.2–65.0)	16,079 (56.2)	26,494 (54.8)	31,011 (64.1)	20,846 (56.4)
Not Current	–	–	–	–	502 (1.4)
Missing, n/N (%)	–	19,743/48,377 (40.8)	0/48,377 (0)	0/48,377 (0)	11,432/48,377 (23.6)

*CI* confidence interval, *CPCSSN* Canadian Primary Care Sentinel Surveillance Networkm, *CTADS* Canadian Tobacco, Alcohol and Drug Survey

^a^*MAR* Missing at Random; assumes missing values are random for all smoking statuses (current, past, never)

^b^*MNAR* Missing not at Random; assumes all current smokers have been documented and missing values are random for never or past smokers

### Method 3 (pattern-matching algorithm)

To validate the accuracy of the pattern-matching algorithm on all available smoking records, all 3295 unique pairs of original Smoking Status and Smoking Value data were manually reviewed from a total of 110,850 smoking records for 39,898 patients. The pattern-matching algorithm correctly assigned a calculated smoking status for 103,935 records (93.8%) (Table 3). In the data review, two new categories were included: ‘Conflicting Information’, which reported on pairs of Status and Value data elements in the same smoking record that were contradicting (e.g. record containing Status = “Current” and Value = “non smoker”); and ‘Not Smoking-related’, which identified records that had been categorized as a smoking record but contained non-smoking information (e.g., blister pack medications). The smoking categories that were most accurately coded by the algorithm were ‘past’ (99.9%), ‘never’ (99.3%),‘not current’ (90.4%), and ‘current’ (83.2%), while the ‘unknown’ category had the least accurate classification rate at 63%. The low accuracy in ‘unknown’ classification results largely because many of the records contain multiple risk factors (such as alcohol use, diet, exercise, etc.). Since the smoking classification algorithm is part of a general risk factor classification algorithm, it is designed to produce a definitive classification when only a single risk factor is identified in a given record.

**Table 3:** Comparison of the pattern-matching algorithm classification to data review

		Data Review (reference standard)							
		Current	Never	Not Current	Past	Unknown	Conflicting Information	Not Smoking-related	Accuracy (%)
**Pattern-Matching Algorithm**	Current	23,858	5	243	298	1562	2688	28	83.2
	Never	0	53,211	32	49	1	274	0	99.3
	Not Current	0	0	2696	2	284	0	0	90.4
	Past	2	1	1	21,691	2	6	0	99.9
	Unknown	236	13	819	282	2479	20	67	63.3

When examining the most recent smoking status for each patient calculated by the pattern-matching algorithm, the proportion of current smokers was slightly lower than in the complete case data (18.2% vs. 21.2%) but was similar to the survey prevalence of 18.9%. Although the algorithm may have more accurately recoded the smoking data, 23.6% of patients were still missing smoking status; however, this is a reduction by 17 percentage points in missingness as compared to the raw, uncoded data of the complete cases.

We conducted a sensitivity analysis to examine whether a different definition for the cohort (at least *three* encounters within the two-year study period, rather than one or more encounters) would elicit differences in the proportion of patients in each smoking status category and in the multiple imputation results. Although fewer patients remained in the new cohort (*n* = 43,375), their demographic and clinical characteristics remained largely the same and no significant differences were found for the multiple imputation.

## Discussion

This study examined post-extraction methods for improving the quality of smoking data in a primary care EMR database. The methodologies explored here differed by their aims and processes – the goal of MI is to improve the completeness of data using other variables in the dataset while also accounting for variability of these data. While the fully conditional multiple imputation successfully eliminated all missing smoking data, there is uncertainty around the accuracy of the smoking status classification. We used a province-specific general population survey to estimate the external validity of the smoking classifications produced by the MI; however, the CTADS survey data have their own limitations, such as a low response rate, small sample size, and non-response bias [13], which means the true prevalence of smoking is still unclear and particularly for adults with hypertension. Thus, the smoking information produced by MI is more appropriate for epidemiological purposes rather than for use in clinical feedback or practice quality improvement.

The rationale for smoking status as MNAR data in the MI was that current smokers are more likely to be captured in the EMR data, given its importance as a cardiovascular risk factor. Method 2 (MNAR) resulted in a lower proportion of current smokers (12.5%) compared to the other methods and survey data, which ranged from 18.2–25.3% (Table 1). It may be possible that for older adults with chronic conditions, emphasis is placed on smoking cessation and therefore, lower proportions of current smokers are observed in this cohort compared to population-level surveys. This finding is similar to a Canadian direct health measures survey, where only 9.9% of females and 14.7% of males treated for hypertension were current smokers; this analysis was focused on a smaller sample of older adults aged 60–79 (*n* = 2111) [14]. Furthermore, the proportion of smokers was found to decline with increasing age in a U.K. primary care database, which was also accompanied by a rising proportion of former smokers as age increased.

The pattern-matching algorithm was designed to use additional information in the smoking record to produce more complete and accurate smoking statuses on an individual level, which is the recommended method for purposes where the accuracy of an individual’s smoking status has greater significance – for example, when using primary care EMR data for practice quality improvement, clinical feedback, or generating a cohort or registry of current smokers. Although this method resulted in reasonably accurate smoking status classifications, the algorithm was still not able to classify 24% of smoking records; further, the category of ‘not current’ is ambiguous and may not be useful information on its own, as this may represent either former or never smokers (though, the number of total records in this category was small). These issues underscore the larger challenges associated with unstructured, inconsistent textual data in primary care EMRs. Any coding or classification that is applied to the CPCSSN data is predicated on the data being present and accurate in the record. The source of missing or inaccurate data can be difficult to identify, as there are numerous places throughout the data flow pathway that can contribute to poor data quality [15]. This includes during point-of-care encounters (patient self-reporting to clinician; poor data entry; lack of structured fields in the EMR to enter smoking information or EMR containing multiple fields where conflicting information is entered), during data extraction, or through data processing (misclassification or errors in the coding algorithm) [7, 15].

Ultimately, a variety of approaches are required to achieve complete, consistent, and accurate information about patient smoking status in EMRs. Any post-extraction approach to data quality improvement relies on complete and accurate data, which therefore means that strategies to support meaningful use of EMRs at the point of care are critical. For example, we found a statistically significant difference in the recording of smoking status attributed to type of EMR system. There are many different EMR products that exist with no formal requirements on the type or location of fields included in the EMR or how the interface appears to the user. Some systems allow for more structured, consistent information to be recorded in an easy-to-navigate way, though some contain more unstructured free text or check-boxes in hard-to-access areas of the chart that can be more difficult for the extraction and interpretation of data. In addition to developing better tools for providers, there have been other examples of interventions to enhance data quality at the practice-level: employing a dedicated data entry clerk was found to improve the quality and usability of EMR data (particularly for smoking status), though the long-term sustainability and scale may not be feasible [16]; implementing data quality feedback tools, reports, and educational training for clinicians have been shown to improve the recording of information in electronic health records [17, 18], as well as reduce variations in quality due to different electronic systems [17]; and lastly, policy-based initiatives, such as meaningful use regulations and financial incentives, have had a positive impact on the recording of smoking status, among other data elements [19, 20]. Even so, data improvement at the point-of-care require more intensive interventions and resources, and therefore post-data extraction strategies should also be pursued. This includes further exploration of the methods described in this paper, as well as more advanced techniques like natural language processing [21].

### Limitations

The first limitation of this study is the lack of a true reference standard from which to assess external validity. By not knowing the true prevalence of smokers (and other smoking statuses) among those with hypertension, we cannot say for certain whether the MI methods are correct from a population-level. The CTADS survey included in the paper reported on the smoking status of individuals in the general Alberta population and was not specific to hypertension, unlike the patient sample here. Other sources were considered but not used for various reasons: the Canadian Community Health Survey (CCHS) and the Canadian Health Measures Survey (CHMS) do not have publicly available smoking rates for people with hypertension. The CCHS describes the proportion of self-reported current (daily or occasional) smokers but not former or non-smokers. The CHMS only includes participants up to the age of 79.

The CPCSSN primary care EMR data also have specific shortcomings. Most importantly, the smoking data within the EMR may be limited in terms of its accuracy and timeliness, which would subsequently impact the results of the MI and pattern matching algorithm. Since smoking status is a dynamic state that can change at any point in time, it is difficult to confirm whether the data present in the EMR are a true reflection of each patient’s current smoking status. We attempted to address this by selecting the most recent smoking status available in the EMR for each patient and found that the majority of this cohort had their most recent smoking status recorded within the previous 2 years.

The CPCSSN data represent a relatively small sample of providers (just over 5% of all family physicians in Alberta [22]) and patients in Alberta (from a provincial population of over 4.3 million in 2019 [23]), and thus may be reasonably but not entirely representative of all providers and patients in the province or elsewhere in Canada. Both the MI and pattern-matching algorithms may perform differently depending on variations between different regional databases and the EMR system from which the data are extracted (e.g., only 5 out of the 11 EMR systems within the CPCSSN database were represented here).

## Conclusion

Multiple imputation and algorithmic pattern-matching are two ways that EMR data can be improved post-extraction, with MI returning complete data and the classification algorithm permitting accuracy estimates. There is potential to further enhance the algorithm using more complex coding with more data points, which would further improve its accuracy and ability to reduce missing patient smoking status. In the future, these methods could also be tested on other risk factor information in a primary care EMR databases, such as alcohol use, diet and exercise.

## Data Availability

The national CPCSSN data are available to approved researchers for a fee; for more information or to submit a Letter of Intent, visit: http://cpcssn.ca/research-resources/ The Alberta-specific CPCSSN data that was used for this analysis are available as two separate data sets through the regional networks (NAPCReN, SAPCReN). Data access procedures and requirements vary by network; contact the corresponding author for more information or visit: http://napcren.ca / http://sapcren.ca

## Abbreviations

BMI: Body mass index
BP: Blood pressure
CI: Confidence interval
CPCSSN: Canadian primary care sentinel surveillance network
CTADS: Canadian tobacco, alcohol, and drugs survey
EMR: Electronic medical record
kg/m^2^: kilograms per metres squared
MAR: missing at random
MI: multiple imputation
MICE: multivariate imputation by chained equations
MNAR: missing not at random
NAPCReN: Northern alberta primary care research network
SAPCReN: Southern alberta primary care research network
SD: Standard deviation
UK: United Kingdom
